# Supplementary material for: Anti-relapse neurons in the infralimbic cortex of rats drive relapse-suppression by drug omission cues
Source: Nat Commun. 2019 Sep 2;10:3934. doi: 10.1038/s41467-019-11799-1 (PMC6718661; doi:10.1038/s41467-019-11799-1)
Supplement: Supplementary file 1 — Supplementary Information [file 41467_2019_11799_MOESM1_ESM.pdf]

**Laque et al. "Anti-relapse neurons in the infralimbic cortex of rats drive relapse-suppression by drug omission cues"**

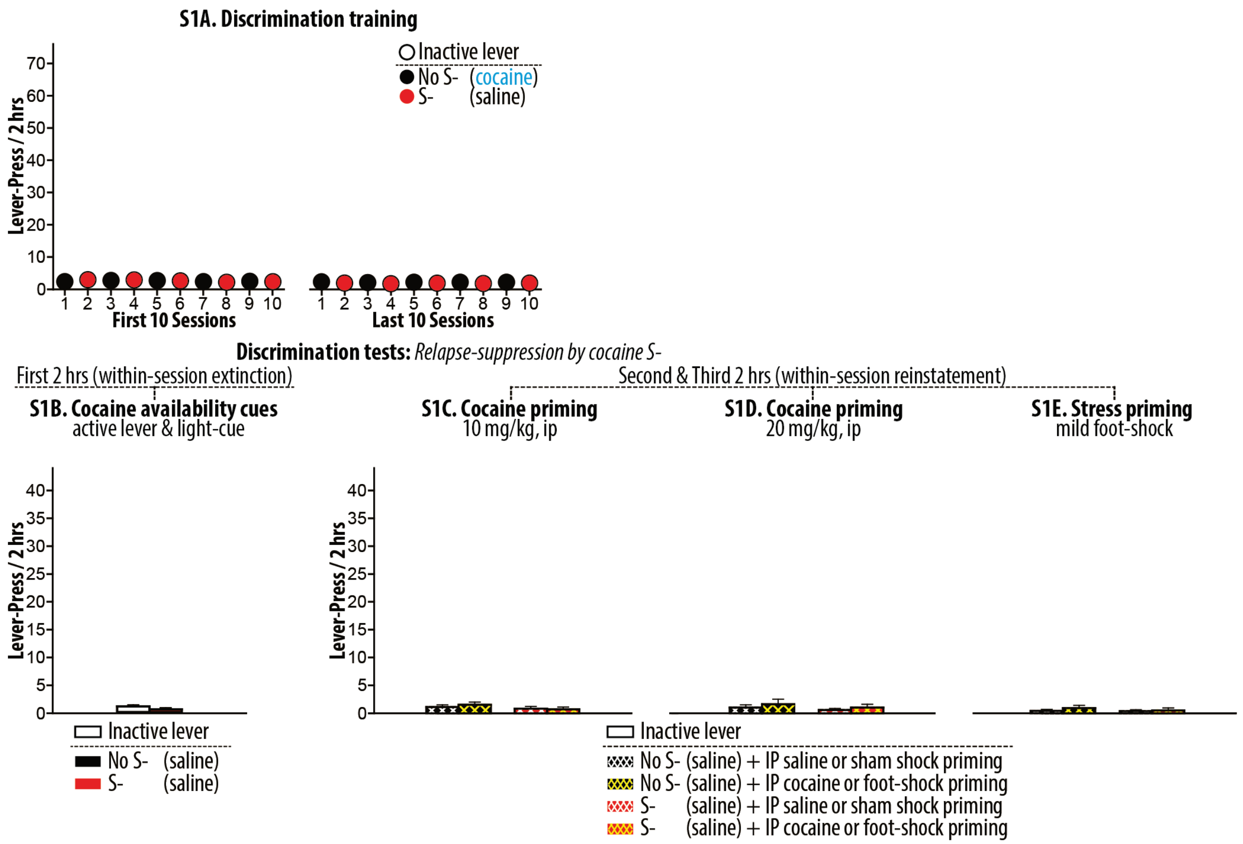


**Supplementary Figure. 1.** The omission cue-induced suppression (OCIS) procedure for cocaine seeking: inactive lever-presses during Discrimination training and Discrimination tests. For all cases, symbols and bars represent group means; horizontal lines represent SEM. **(a)** Responses during the first and last 10 days of the Discrimination training. **(b)** Responses during the first 2-hour block of the Discrimination test to determine the anti-relapse action of cocaine S- against cocaine availability cues (active lever and light-cue). N=33. **(c, d)** Responses during the second and third 2 hour-blocks of the Discrimination test to determine the anti-relapse action of cocaine S- against cocaine priming (10 and 20 mg/kg, IP). N=12 and 10 each. **(e)** Responses during the second and third 2 hour-blocks of Discrimination test to determine the anti-relapse action of cocaine S- against stress priming (mild electric foot-shock, intermittent, 10 min, 0.8 mA). N=11.

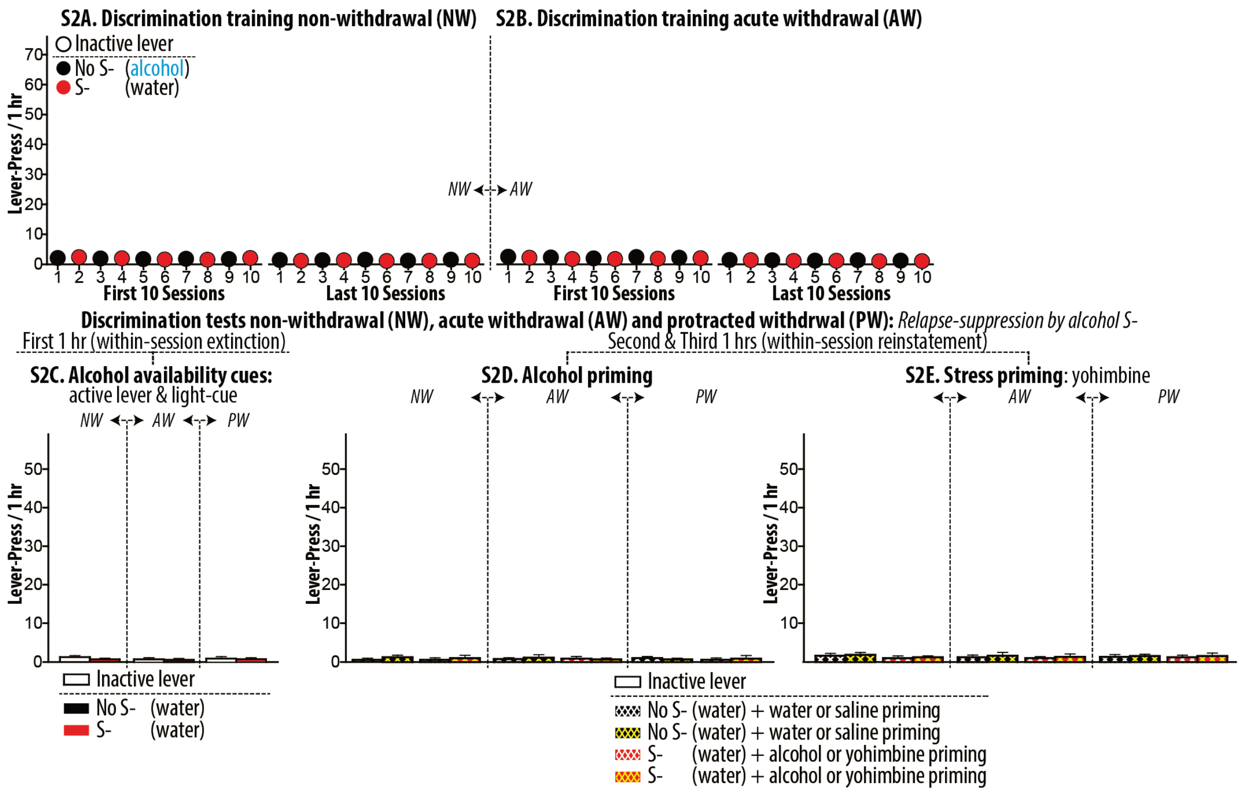


**Supplementary Figure. 2.** The omission cue-induced suppression (OCIS) procedure for alcohol seeking: inactive lever-presses during Discrimination training and Discrimination tests. For all cases, symbols and bars represent group means; horizontal lines represent SEM. **(a, b)** Responses during the first and last 10 days of the Discrimination training under non-withdrawal (NW) and acute withdrawal (AW) conditions. N=25. **(c)** Responses during the first 1-hour block of Discrimination test to determine the anti-relapse action of alcohol S- against alcohol availability cues (active lever and light-cue) under NW, AW and protracted withdrawal (PW) conditions. N=25. **(d)** Responses during the second and third 1-hour blocks of the Discrimination test to determine the anti-relapse action of alcohol S- against alcohol priming (20%, v/v, 0.2 ml, oral) under NW, AW and PW conditions. N=12. **(e)** Responses during the second and third 1 hour-blocks of the Discrimination test to determine the anti-relapse action of alcohol S- against stress priming (yohimbine, 0.75 mg/kg, IP). N=13.

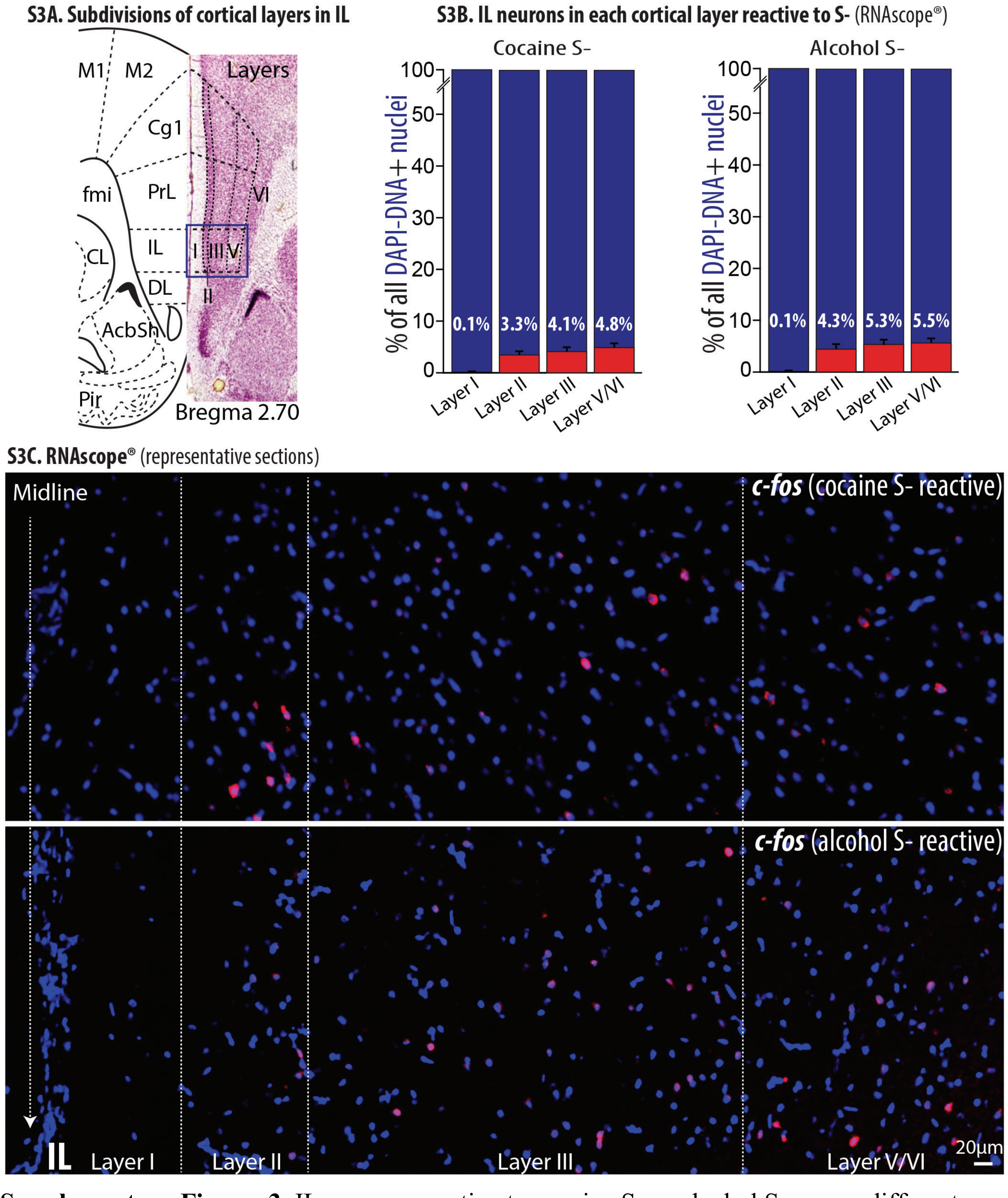


**Supplementary Figure. 3.** IL neurons reactive to cocaine S- or alcohol S- across different cortical layers. Data represent group means and SEM. For statistical analyses, total numbers of nuclei per mm$^2$ in each cortical layer that satisfied the criterion for "S- reactive" phenotype were used. For graphic representations, percentages of "S- reactive" phenotype in different cortical layers were used. **(a)** Target sites and layers: I, II, III, and V/VI (4 levels: layer V and VI were analyzed jointly; no layer IV in rats). **(b)** Omission cue-reactive neural phenotype in IL as indicated by RNAscope® targeting *c-fos* as a marker for "S- reactive" nuclei. Each nucleus was

identified by DNA-staining DAPI. N=7 (cocaine S-) and 8 (alcohol S-). Two-way mixed ANOVA: Layer ($F_{(3,39)}$ = 23.03, P<0.001) but not Group ($F_{(1,13)}$ = 0.48, NS). **P*<0.001 versus layer I. Tukey HSD test. **(c)** Representative sections of IL from rats trained for cocaine S- and alcohol S-. ***Note:** These analyses were based on additionally processed brain sections from the same subjects included in the results depicted in Fig 3 (7 rats for cocaine S- and 8 rats for alcohol S-). This resulted in minor variations in S- reactivity (the percentages of *c-fos*+ nuclei within DAPI+ nuclei) within the same Group (cocaine S- or alcohol S-) between the results depicted in Fig 3 and Supplementary Fig 3.

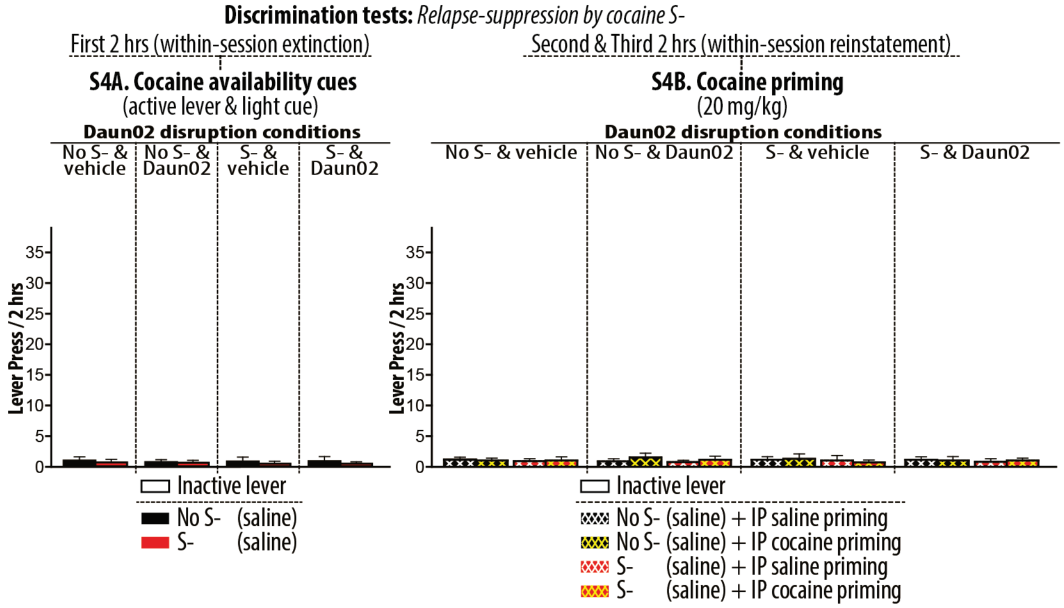


**Supplementary Figure. 4.** OCIS procedures for functional characterization of omission cue-activated neurons via Daun02 disruption: inactive lever-presses during the Discrimination training and the Discrimination tests. For all cases, bars represent group means; horizontal lines represent SEM. **(a)** Responses during the first 2-hour block of the Discrimination test (within-session extinction) to determine anti-relapse action of cocaine S- against cocaine availability cues (active lever and light-cue). N=7,7,8,8. **(b)** Responses during the second and third 2 hour-blocks of Discrimination test (within-session reinstatement) to determine anti-relapse action of cocaine S- against cocaine priming (20 mg/kg, IP). N=7,7,8,8.
